# Assessment of MRI-Based Attenuation Correction for MRI-Only Radiotherapy Treatment Planning of the Brain

**DOI:** 10.3390/diagnostics10050299

**Published:** 2020-05-14

**Authors:** Iiro Ranta, Jarmo Teuho, Jani Linden, Riku Klén, Mika Teräs, Mika Kapanen, Jani Keyriläinen

**Affiliations:** 1 Department of Physics and Astronomy, University of Turku, Vesilinnantie 5, FI-20014 Turku, Finland; jani.keyrilainen@tyks.fi; 2 Department of Medical Physics, Turku University Hospital, Hämeentie 11, FI-20521 Turku, Finland; jarmo.teuho@tyks.fi (J.T.); mika.teras@tyks.fi (M.T.); 3 Department of Oncology and Radiotherapy, Turku University Hospital, Hämeentie 11, FI-20521 Turku, Finland; 4 Turku PET Centre, University of Turku and Turku University Hospital, Kiinamyllynkatu 4-8, FI-20521 Turku, Finland; jjlind@utu.fi (J.L.); riku.klen@tyks.fi (R.K.); 5 Department of Mathematics and Statistics, University of Turku, Vesilinnantie 5, FI-20014 Turku, Finland; 6 Institute of Biomedicine, University of Turku, Kiinamyllynkatu 10, FI-20014 Turku, Finland; 7 Department of Medical Physics, Medical Imaging Center, Tampere University Hospital, Teiskontie 35, FI-33521 Tampere, Finland; mika.kapanen@pshp.fi; 8 Department of Oncology, Unit of Radiotherapy, Tampere University Hospital, Teiskontie 35, FI-33521 Tampere, Finland

**Keywords:** MRI-only, brain radiotherapy, RTP, PET–MRI, MRAC

## Abstract

Magnetic resonance imaging-only radiotherapy treatment planning (MRI-only RTP) and positron emission tomography (PET)–MRI imaging require generation of synthetic computed tomography (sCT) images from MRI images. In this study, initial dosimetric evaluation was performed for a previously developed MRI-based attenuation correction (MRAC) method for use in MRI-only RTP of the brain. MRAC-based sCT images were retrospectively generated from Dixon MR images of 20 patients who had previously received external beam radiation therapy (EBRT). Bone segmentation performance and Dice similarity coefficient of the sCT conversion method were evaluated for bone volumes on CT images. Dose calculation accuracy was assessed by recalculating the CT-based EBRT plans using the sCT images as the base attenuation data. Dose comparison was done for the sCT- and CT-based EBRT plans in planning target volume (PTV) and organs at risk (OAR). Parametric dose comparison showed mean relative differences of <0.4% for PTV and <1.0% for OARs. Mean gamma index pass rates of 95.7% with the 2%/2 mm agreement criterion and 96.5% with the 1%/1 mm agreement criterion were determined for glioma and metastasis patients, respectively. Based on the results, MRI-only RTP using sCT images generated from MRAC images can be a feasible alternative for radiotherapy of the brain.

## 1. Introduction

Magnetic resonance imaging-only radiotherapy planning (MRI-only RTP) and hybrid positron emission tomography (PET)–MRI are emerging applications. Electron density information typically acquired from computed tomography (CT) images is a prerequisite for RTP and attenuation correction in PET. However, MR images represent tissue relaxation times and proton density information.

To solve this challenge, several methods to produce synthetic CT (sCT) images have been developed for both MRI-only RTP and MRI-based attenuation correction (MRAC) [1,2,3,4,5]. Thus, the methods that produce accurate attenuation correction on PET–MRI could potentially be applied in MRI-only RTP and vice versa. If a single sCT generation method could be used in both applications, there would be no need to use separate sequences or processing pipelines for producing sCT between different systems and modalities. However, a thorough evaluation of the method robustness in patients with brain tumors and presence of MRI contrast agents should be performed.

Previous research in the field of MRI-only RTP of the brain has shown that, on average, the effect of sCT- vs. CT-based dose calculation on the total dosimetric uncertainty is <2%. This scale of accuracy has been reported for planning target volumes (PTV) using various sCT generation methodologies [6,7,8,9].

In addition to dose calculation uncertainty, the systematic registration error between planning CT and MRI images contributes to total uncertainty present in the RTP process. In the head region, average registration errors <2 mm have been reported [10]. The use of MRI-only RTP workflow eliminates this systematic registration error and may therefore reduce the total dosimetric uncertainty induced during the RTP process [11].

One of the challenges typical for sCT generation methods is the accurate segmentation of the bone when anatomical abnormalities and MRI contrast agents are present. The anatomical abnormalities can be caused by medical interventions, primary tumor growth such as in gliomas, or growth of secondary tumors due to metastases [12]. Commonly used gadolinium-based contrast agents lower the tissue T1 relaxation time, resulting in changes in the MR signal intensity. Thus, the definition of anatomical structures by segmentation-based approaches might be affected by the presence of gadolinium-based contrast agents [13]. Therefore, it is valuable to reevaluate the accuracy of the MRAC methods in more challenging patient populations undergoing MRI-based RTP of the brain.

In previous studies, zero echo time (ZTE) pulse sequences have been used to successfully generate sCT images that can be used for accurate MRAC and MRI-only RTP of the brain [14]. Similar evaluations have not yet been performed for other sequence types. Furthermore, the effect of contrast agents in sCT generation and their effects in radiation therapy (RT) plan quality have not been studied extensively in an individual study, although methods based on deep learning have been successfully used to generate sCTs from contrast-enhanced images [15,16].

In this retrospective study, we investigated the application of a MRAC method to RTP of brain tumors with regard to dose calculation and bone segmentation accuracy. The dose calculation accuracy was assessed by direct dosimetric comparison, gamma evaluation, and statistical analysis for PTV and organs at risk (OAR) volumes. Bone segmentation accuracy was determined by analysis of bone volume and Dice similarity coefficient (DSC). The MRAC method has previously been assessed in PET–MRI of the brain concerning both quantitative and visual accuracy of [18F]-fluorodeoxyglucose ([18F]-FDG) PET images [17,18].

## 2. Materials and Methods

### 2.1. Patient Cohort

Patient cohort for the current study consisted of 20 patients with either glioma (*n* = 10) or brain metastases from various primary cancers (*n* = 10). All patients received EBRT using 6 MV photon beams at Turku University Hospital (Turku, Finland). All participants gave written informed consent for their participation and the study was approved by the Ethical Committee of the Hospital District of Southwest Finland (reference code: Dnro 116/1801/2017, approval date: 21 November 2017). During the RTP imaging sessions, the head fixation of all patients was done using thermoplastic masks (Orfit Industries N.V., Wijnegem, Belgium). Clinical details about the patient cohort are provided in Table 1. More detailed information about the patient cohort is provided in Appendix A, Table A1.

### 2.2. MRI and CT Data Acquisition

MRI data acquisition for all patients was performed with Philips Ingenia 1.5T HP MR-RT (Philips Medical Systems International B.V., Best, The Netherlands) during a routine MRI simulation session. T1-weighted 3D mDixon images acquired after gadolinium contrast agent injection were used as the base image data for sCT generation. Due to patient fixation, two flex coils and a 16-channel anterior body coil were used to record the MRI data instead of a diagnostic head coil. The essential imaging parameters are presented in Table 2.

The CT images for all patients were obtained with Toshiba Aquilion LB (Toshiba Corp., Tokyo, Japan) during a normal CT simulation session. The images were obtained using 120 kV tube voltage, 50 mA tube current, 1.0 × 1.0 mm^2^ reconstruction resolution, and slice thickness of 2.0 mm for glioma patients and 1.0 mm for metastasis patients.

### 2.3. Generation of sCT Images for MRI-Only RTP

sCT images were created based on the MRAC method [17,18], which enables creation of MRI-based attenuation maps for up to six tissue classes and has been shown to perform with good accuracy regarding PET quantification. The method is simple, fast, and straightforward to apply across different PET-MRI and MRI-only RTP systems. Essentially, the method is based on segmentation of MRI images to different tissue classes. The MRI images can be either T1- or T2-weighted.

The current method included six tissue classes with individual Hounsfield unit (HU) values determined based on the literature, including cortical bone (942 HU), air (−1000 HU), grey matter (41 HU), white matter (25 HU), and cerebrospinal fluid (15 HU). The segmentation engine was based on the New Segment implemented in SPM8 (The Wellcome Centre for Human Neuroimaging, UCL Queen Square Institute of Neurology, London, UK), modified for Segment in SPM12 in this study [19].

The processing pipeline was implemented in MATLAB 2015b (MathWorks Inc., Natick, MA, USA) and took MRI images as input, performed segmentation to six different classes in the Segment function of SPM12, and created binary masks of different tissue classes, which were then used to form the final sCT. Finally, the sCT images were converted to DICOM (digital imaging and communications in medicine) format to enable them to be exported to the treatment planning system (TPS). During data processing, the MRI and CT images were registered and resolution-matched using the default registration functions in SPM12.

### 2.4. Segmentation Evaluation for sCT Images

Segmentation accuracy was assessed by DSC analysis, while the bone delineation accuracy was determined by measuring the bone volume in the sCT versus CT. For determining the segmentation accuracy, registered sCT and CT were converted into binary masks representing the skull bones using a predefined threshold of 300 HU as defined in the report by Aasheim et al. [20]. Thereafter, the DSC was calculated as follows [21]:

(1) $$\mathrm{DSC}=\frac{2|A\;\cap \;B|}{\left|A|+|B\right|}$$

where A represents the binary skull mask from the sCT, while B represents the binary skull mask from the CT.

The bone volume was determined by calculating the average amount of bone voxels in each image slice in the sCT and CT. The average amount of bone voxels was assessed as a sum of bone voxels in each individual slice. Finally, to compare the differences between the CT and sCT HU values, the mean absolute error (MAE) for HU values was calculated for all patients within the body volume from the crown to cranial base using a MATLAB script.

### 2.5. Dose Calculation Accuracy of sCT Images for RTP

Dose calculation accuracy of the generated sCT images was evaluated in Eclipse TPS (version 15.6., Varian Medical Systems Finland Oy, Helsinki, Finland). The generated sCT images were imported to TPS, and the sCT-based RT plans were generated by copying the existing CT-based RT plans over to the corresponding sCT images. Volumetric doses were then recalculated for the sCT-based RT plans using identical RTP parameters.

Dose calculation accuracy of the sCT-based RT plans was assessed by comparing multiple dose–volume histogram (DVH) parameters according to the recommendations presented in the ICRU 83 report [22] for doses of PTV and OAR volumes. To improve interpatient comparability, the OAR volume was defined as the volume of tissue contained within 2 cm from the PTV outer edge. Individual dose differences of DVH parameters were determined as a relative dose difference of sCT plan’s DVH values compared to corresponding CT plan’s values, which can be expressed by the following equation:

(2) $$\Delta D\left(V\right)=\frac{D_{\mathrm{sCT}}\left(V\right){-D}_{\mathrm{CT}}\left(V\right)}{D_{\mathrm{CT}}\left(V\right)}$$

where ΔD(V) is the relative dose difference for certain volume on the DVH, and D_CT_(V) and D_sCT_(V) are the calculated doses for the volume in CT- or sCT-based RT plans, respectively.

Statistically significant DVH dose differences between CT- and sCT-based RT plans were evaluated by performing a Wilcoxon signed-rank test for each DVH parameter. Statistical analyses of DVH differences between glioma and metastasis groups were done using the Mann–Whitney *U* test.

The mutual correspondence of sCT- and CT-based RT plans was further evaluated by performing a global 3D gamma analysis. Both RT plans and the registration data were exported from the TPS, and gamma analysis was performed using an open-source Plastimatch image computation software (version 1.8.0, Plastimatch.org). The gamma analyses were performed with dose difference and distance-to-agreement criteria of 2%/2 mm and the more restrictive 1%/1 mm, with the dose threshold set to >10% of the maximum dose and the maximum gamma value set to default value of 2. Statistical analysis of gamma pass rates between glioma and metastasis groups was performed using the Mann–Whitney *U* test for each dose difference and distance-to-agreement criterion.

## 3. Results

**Figure 1:**
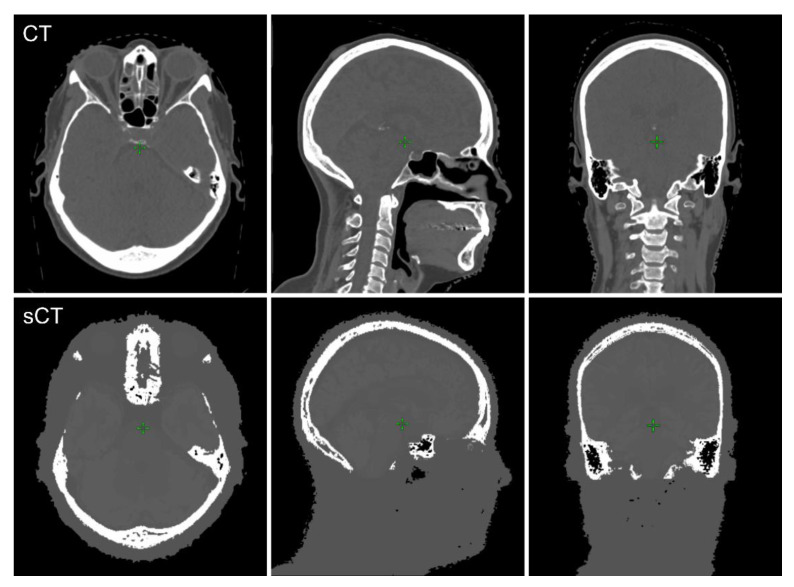
Case example of the CT (top) and generated sCT (bottom) image pairs in transversal, sagittal, and coronal directions. (CT: computed tomography, sCT: synthetic computed tomography).

Generation of sCT images was successful for all 20 patients. A comparison between sCT and CT image quality is illustrated in Figure 1.

### 3.1. Bone Segmentation and Comparison of HU Values

**Figure 2:**
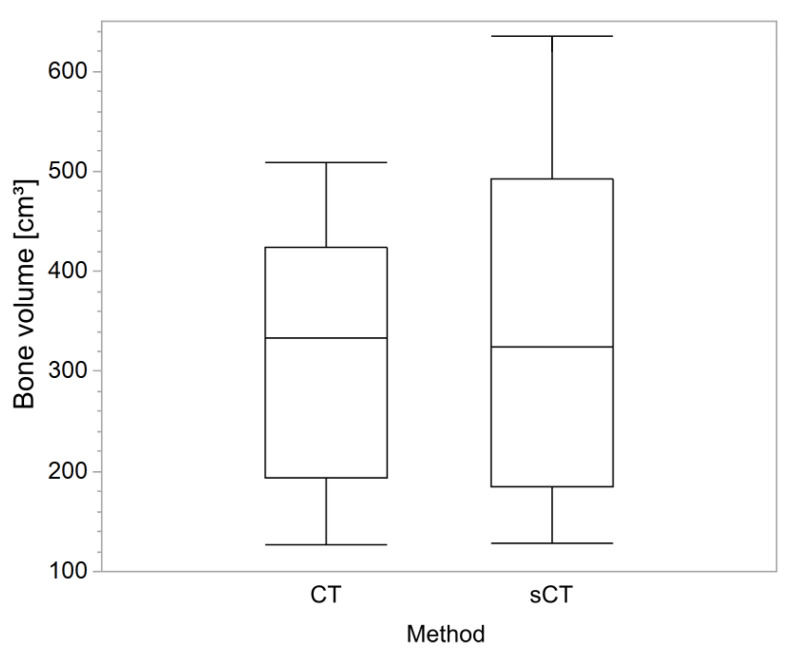
Boxplot of bone volume comparison results between CT and sCT images. The boxes represent one standard deviation from the mean. The whiskers indicate 95% confidence interval from the mean (CT: computed tomography, sCT: synthetic computed tomography).

Bone volumes, DSC values, and MAE values, presented in Figure 2 and Table 3, were successfully determined for all patients. On average, the determined bone volumes were smaller in the glioma group compared with the metastasis group. There was no statistical significance in the relative bone volume differences between the glioma and metastasis groups (*p* = 0.13). The mean DSC and MAE values were similar for both the glioma and metastasis groups with no statistically significant differences (*p* ≥ 0.27).

### 3.2. Dosimetric Comparison

**Figure 3:**
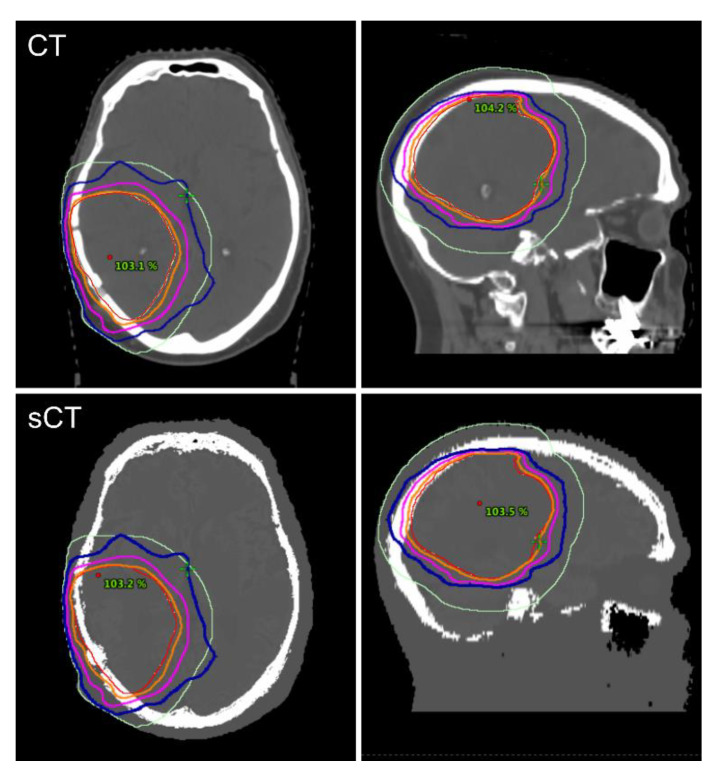
Case example of the RTP comparison between CT- and sCT-based RT plans of a glioma patient. Isodose levels of 90% (orange), 70% (magenta), and 50% (blue) are visualized together with PTV (red) and OAR (light green) contours in transversal and sagittal planes (RTP: radiotherapy treatment planning, CT: computed tomography, sCT: synthetic computed tomography, RT: radiotherapy, PTV: planning target volume, OAR: organs at risk).

Dosimetric comparison was successful for all 20 patients. A comparison between sCT- and CT-based RT plans is illustrated in Figure 3.

**Figure 4:**
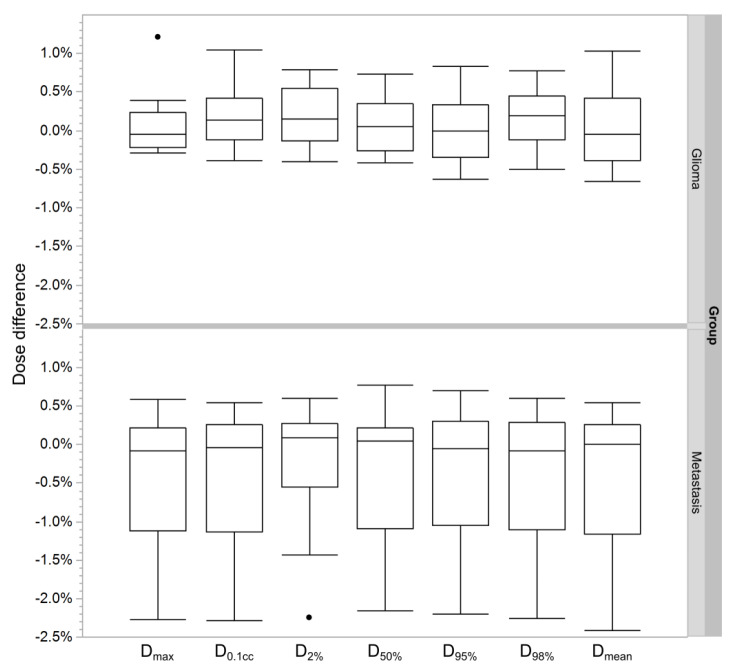
Parametric DVH comparison results for the glioma (top) and metastasis (bottom) patient groups with outlier results included as dots. The boxes represent one standard deviation from the mean. The whiskers indicate 95% confidence interval from the mean (DVH: dose–volume histogram, D: dose).

The mean absolute dose differences for all DVH parameters, presented in Figure 4 and Table 4, were found to be ≤0.4% (0.9%) in both patient groups for the PTV, while the mean absolute difference was ≤1.0% (3.5%) for the OARs. One outlier was found in the metastasis patient group, where the DVH dose differences were ≥2.2% for all DVH parameters of both PTV and OARs.

Statistical evaluation between glioma and metastasis patient groups did not reveal significant differences for any of the determined DVH parameters (*p* ≥ 0.21). Furthermore, no statistically significant differences between sCT and CT doses were found for any of the reported DVH parameters in the glioma or metastasis patient groups (*p* ≥ 0.14).

### 3.3. Gamma Analysis

Gamma analysis results, presented in Figure 4 and Table 5, showed gamma index pass rate of >95% pass with 1%/1 mm criterion for the metastasis patient group and >95% pass with 2%/2 mm criterion for the glioma patient group. One outlier result was found in the metastasis patient group with pass rate of <90% pass with 1%/1 mm criterion. The outlier result was observed with the same patient that was also identified as an outlier in the DVH analysis. The statistical analysis showed a significant difference in pass rates of the glioma and metastasis patient groups with the 2%/2 mm criterion (*p* = 0.04).

## 4. Discussion

The aim of this assessment study was to perform a preliminary dosimetric evaluation for sCT images generated using a previously developed MRAC method for MRI-based RTP of the brain. The evaluation was performed by comparing the relative dosimetric differences between the PTV and OAR regions in the CT and sCT images. The dose difference and distance-to-agreement criteria were also studied by performing a global 3D gamma analysis. Multiple anatomical variations, tumor types, and tumor locations were included in the patient cohort, ranging from large gliomas to individual brain metastases with PTV volume of <1 cm^3^.

Based on the results, neither contrast agents nor anatomical deformities hindered the delineation of bone or other tissue classes. Thus, the segmentation seems robust even in the presence of anatomical deformation and contrast agents. Further increase in accuracy would be achieved by implementing an accurate method to differentiate bone, air, and a mix of soft tissue in the nasal sinus region. The resulting RT plan quality was also found comparable to sCT-based RTPs generated from non-contrast-enhanced MRIs in other recent studies that adopted machine learning- and deep learning-based approaches for sCT generation [15,16]. The current results indicate that sufficient brain RT plan accuracy could also be achieved using the studied MRAC method while using contrast-enhanced images for sCT generation.

The relative dosimetric differences of sCT- vs. CT-based plans were ≤0.4% for all compared DVH parameters in the PTV regions of both patient groups. Based on the statistical evaluations, the dosimetric performance of the MRAC model in the PTVs did not differ between glioma and metastasis patients. The results indicate better dosimetric accuracy than previous studies using Dixon-based sequences, where differences of 1.4% were reported for clinical target volume alone when using protons [6]. The MRAC model reached similar dosimetric accuracy to those of voxel-based sCT methods with ultrashort echo time (UTE) sequences in the brain region [8,9] that have reported absolute D_mean_ differences of ≤0.3% for the PTV.

Acceptance criteria of <2% dose difference in PTV coverage for 95% of head and neck patients has been proposed previously for the reliable clinical use of MRI-only RTP techniques [23]. The gamma analysis results demonstrated that this criterion could be met by a majority of the patient cohort in both glioma and metastasis patient groups, with mean pass rates of 95.7% and 99.9%, respectively. Furthermore, the mean pass rate of 96.5% in the metastasis patient group with 1%/1 mm agreement criterion gives an indication that the studied MRAC method could also be a feasible sCT generation method in MRI-based stereotactic RTP of the brain, where the accuracy requirements are stricter than elsewhere. Similar mean pass rates were also reported in [24] for metastasis patients (97% pass rate using a threshold of >10% of the prescribed dose) while using a convoluted neural network approach for sCT generation. It is important to note that the comparability of the current results to the literature is limited as the essential parameters used during gamma analysis are not routinely reported, as recommended by Hussein et al. [25]. In the current study, a global 3D gamma analysis was used to maximize the comparability of results.

While there was a statistical difference in the pass rate with 1%/1 mm criterion between the glioma and metastasis groups, this can be expected due to larger PTV volumes of gliomas compared with those of metastases. Due to the increase in PTV, the volume evaluated during gamma analysis may extend to regions with greater tissue inhomogeneity and registration error, thus increasing the dosimetric uncertainty and lowering the gamma index pass rate of glioma patients compared with metastasis patients.

One outlier with >2% absolute DVH dose differences within PTV and OAR was found in the metastasis patient group. The probable reason for the outlier result was the overestimation of body outline in the sCT image compared with the CT image, which led to underestimated radiation dose in the PTV. It is also possible that the use of thermoplastic fixation masks had an effect on body surface definition. This outlier result was also reflected in the gamma analysis results as the gamma pass rate with 1%/1 mm and 2%/2 mm criteria were significantly lower in comparison to other patients. Generally, the difference in body outline has a significant effect on dose calculation accuracy as an increase of 1 cm in body outline may result in >3% relative dose differences [26]. This effect is further amplified in low dose regions, where the absolute dose differences are small.

The current SPM12 template only included the skull region by default. In future studies, the generation of a custom template [27] from the MRI sequence used for RTP would increase the bone delineation accuracy, decrease the MAE, and improve tissue segmentation in nasal cavity. This should reduce the over- and underestimations in outlier patients. As our purpose now is to study the application of the methodology in its baseline state, in the future, we will investigate the inclusion of such a custom template on the head and neck area as an extension of the current study.

The assessment of dose calculation accuracy was done by registering the sCT images with the CT images. The registration error may have an effect on the results on areas further from the PTV, where the registration errors are more likely to be significant and the absolute dose differences are smaller than within the PTVs. Furthermore, the effect of different RT techniques, e.g., intensity-modulated RT vs. coplanar volumetric-modulated arc therapy vs. noncoplanar techniques, on dose calculation accuracy was not investigated in this pilot study.

The current study did not evaluate the effect of using sCT- vs. CT-based digitally reconstructed radiographs (DRR) in the verification of patient positioning. In previous studies for voxel-based sCT generation methods in the brain region, the positioning verification accuracy of sCT-based DRRs has been found comparable to CT-based DRRs with reported differences of <1 mm [28,29]. Further optimizations of the studied MRAC model with MRI-based RTP aspect in mind could be done to improve the quality of sCT-based DRRs. As a result, validation of positioning verification accuracy could be performed for sCT- vs. CT-based DRRs in future studies.

## 5. Conclusions

Based on the results of this study, the sCT images generated using a MRAC method could be feasible for planning and dose calculation of MRI-based RTP of the brain. The results enable further development and investigation of the MRAC method by creating a custom template for increasing the accuracy of bone delineation, improving segmentation in the nasal cavity, and extending the assessment to the head and neck area.

## Figures and Tables

**Table 1:** Glioma and metastasis patient characteristics (SD: standard deviation, GTV: gross tumor volume, PTV: planning target volume, OAR: organs at risk).

Structure	Glioma	Metastasis
	Mean Volume (SD) [Range] [cm^3^]	
GTV	50.0 (28.8) [14.2–112.3]	4.3 (5.2) [0.1–16.4]
PTV	267.3 (82.1) [132.8–373.3]	8.6 (8.8) [0.3–28.8]
OAR	577.7 (126.9) [357.9–764.0]	131.8 (57.1) [59.9–258.9]

**Table 2:** Imaging parameters for MRI data acquisition (MRI: magnetic resonance imaging, TE: echo time, TR: repetition time, BW: bandwidth, FFE: fast field echo).

Sequence	Acq. Matrix [mm^3^]	Recon. Matrix [mm^3^]	TE1/TE2 [ms]	TR [ms]	Flip Angle [°]	BW [Hz]	Scan Time [min:s]
T1 3D FFE mDixon	1.1 × 1.1 × 1.4	0.49 × 0.49 × 1.00	2.0/4.4	6.8	20	481.5	5:38

**Table 3:** Mean results, respective standard deviations, and ranges for Dice similarity coefficient (DSC) and bone volume (V) analysis for the glioma and metastasis patient groups.

Variable	Glioma	Metastasis
V_CT, bone_ [cm^3^]	220.4 (81.4) [126.6–443.1]	410.7 (59.7) [305.3–508.1]
V_sCT, bone_ [cm^3^]	227.4 (127.2) [128.1–585.5]	465.4 (98.1) [280.9–634.8]
ΔV_bone_ [%]	3.1 (19.5) [−34.5–32.1]	12.5 (11.8) [−8.0–26.8]
DSC_bone_ []	0.8 (0.1) [0.62–0.86]	0.8 (0.02) [0.80–0.86]
MAE [HU]	142.2 (15.4) [114.4–166.7]	139.7 (11.8) [114.4–166.6]

**Table 4:** Mean results, respective standard deviations, and ranges of the parametric DVH dose comparisons for 10 glioma and 10 brain metastasis patients in PTV and OARs (DVH: dose–volume histogram, PTV: planning target volume, OAR: organs at risk, SD: standard deviation, D: dose).

DVH Parameter	Glioma		Metastasis	
	PTV	OAR	PTV	OAR
	Mean Dose Difference (SD) [Range] [%]			
D_max_	0.1 (0.5) [−0.6–1.3]	0.2 (1.5) [−1.8–4.0]	−0.4 (0.9) [−2.4–0.5]	−0.4 (0.9) [−2.1–0.5]
D_0.1cc_	0.1 (0.4) [−0.1–1.2]	0.3 (1.0) [−1.8–2.0]	−0.4 (0.9) [−2.2–0.6]	−0.3 (0.8) [−2.0–0.6]
D_2%_	0.2 (0.4) [−0.4–1.1]	0.3 (0.5) [−0.2–1.2]	−0.4 (0.9) [−2.3–0.6]	−0.5 (1.1) [−2.9–0.8]
D_50%_	0.2 (0.4) [−0.4–0.8]	0.2 (0.4) [−0.4–1.0]	−0.3 (0.9) [−2.3–0.6]	−0.3 (1.4) [−3.3–1.6]
D_95%_	0.1 (0.4) [−0.4–0.7]	−1.2 (2.3) [−5.6–1.7]	−0.3 (0.9) [−2.2–0.8]	−0.6 (1.8) [−3.6–2.6]
D_98%_	0.04 (0.4) [−0.6–0.8]	−1.3 (2.0) [−4.9–2.0]	−0.4 (0.9) [−2.2–0.7]	1.0 (3.5) [−3.0–8.3]
D_mean_	0.2 (0.4) [−0.5–0.8]	0.1 (0.5) [−0.6–1.0]	−0.4 (0.9) [−2.3–0.6]	−0.6 (1.2) [−3.7–1.0]

**Table 5:** Mean gamma index pass rate percentages, respective standard deviations, and ranges for 10 glioma and 10 brain metastasis patients with different dose difference and distance-to-agreement criteria (SD: standard deviation).

Agreement Criterion	Glioma Group	Metastasis Group
	Mean Pass Rate (SD) [Range] [%]	
1%/1 mm	90.7 (3.6) [85.6–95.0]	96.5 (4.7) [84.3–100.0]
2%/2 mm	95.7 (0.9) [93.9–96.8]	99.9 (0.3) [99.1–100.0]

## Appendix A

**Table A1:** Characteristics of individual patients and details of external beam radiotherapy (F: female, M: male, GTV: gross tumor volume, PTV: planning target volume, OAR: organs at risk, Fx: fractions, SRT: stereotactic radiation therapy, VMAT: volumetric-modulated arc therapy).

Patient	Sex/Age (F/M)/[y]	Group	GTV Volume [cm^3^]	PTV Volume [cm^3^]	OAR Volume [cm^3^]	Prescribed Dose [Fx × Gy]	Treatment Technique
1	F/71	Metastasis	0.1	0.3	59.9	6 × 5	SRT
2	F/67	Glioma	15.5	149.1	440.1	30 × 1.8	VMAT
3	M/68	Glioma	79.9	280.3	709.9	13×3	VMAT
4	M/74	Glioma	35.0	268.5	549.4	33 × 1.8	VMAT
5	F/68	Glioma	56.0	369.2	622.9	30 × 2	VMAT
6	M/81	Glioma	51.3	289.1	541.7	13 × 3	VMAT
7	F/73	Glioma	112.3	373.3	648.6	13 × 3	VMAT
8	M/55	Metastasis	2.2	4.1	115.5	3 × 9	SRT
9	M/78	Metastasis	4.2	6.4	144.5	4 × 5	SRT
10	F/77	Metastasis	6.7	9.2	136.0	9 × 3	SRT
11	M/77	Metastasis	1.1	1.9	91.2	1 × 20	SRT
12	F/42	Glioma	27.4	132.8	357.9	28 × 1.8	VMAT
13	F/37	Glioma	43.5	266.9	698.2	33 × 1.8	VMAT
14	F/64	Metastasis	0.1	0.4	62.0	1 × 20	SRT
15	F/72	Metastasis	2.6	4.1	117.9	5 × 7	SRT
16	F/70	Metastasis	5.1	9.7	136.7	5 × 6	SRT
17	M/63	Metastasis	16.4	20.6	195.6	3 × 9	SRT
18	M/45	Metastasis	13.0	28.8	258.9	6 × 5	SRT
19	M/50	Glioma	64.7	352.7	764.0	30 × 2	VMAT
20	M/58	Glioma	14.2	191.5	444.3	30 × 2	VMAT

**Table A2:** Bone segmentation results of individual patients. (V: volume, DSC: Dice similarity coefficient).

Patient	V_CT, bone_ [cm^3^]	V_sCT, bone_ [cm^3^]	ΔV_bone_ [%]	DSC_bone_ []
1	431.0	412.8	−4.2	0.83
2	183.9	218.7	18.9	0.83
3	443.1	585.5	32.1	0.86
4	229.5	268.5	17.0	0.86
5	238.2	229.3	−3.7	0.72
6	185.5	139.0	−25.1	0.75
7	164.6	167.8	1.9	0.83
8	500.5	634.8	26.8	0.82
9	362.3	418.8	15.6	0.86
10	377.1	441.9	17.2	0.82
11	448.0	543.9	21.4	0.83
12	243.5	224.0	−8.0	0.79
13	193.1	172.8	−10.5	0.82
14	372.0	367.9	−1.1	0.86
15	398.3	493.0	23.8	0.84
16	403.8	489.4	21.2	0.81
17	305.3	280.9	−8.0	0.80
18	508.1	570.4	12.3	0.85
19	195.6	128.1	−34.5	0.62
20	126.6	140.0	10.6	0.80
